# Six years of facial trauma care: an epidemiological analysis of 355 cases

**DOI:** 10.1590/S1808-86942010000500006

**Published:** 2015-10-22

**Authors:** Thiago Bittencourt Ottoni Carvalho, Launa Renata Londero Cancian, Caroline Gabriele Marques, Vânia Belintani Piatto, José Victor Maniglia, Fernando Drimel Molina

**Affiliations:** 1 Third-year medical resident in ENT and Head and Neck Surgery - FAMERP; 2 Third-year medical resident in ENT and Head and Neck Surgery - FAMERP; 3 PhD; Head of the Dental Service of the ENT-HNS Department - FAMERP; 4 PhD in Health Sciences, Adjunct Professor - Department of ENT-HNS - FAMERP; 5 Associate Professor - Department of ENT-HNS - FAMERP; 6 PhD, Adjunct Professor - Department of ENT-HNS - FAMERP. Medical School of São José do Rio Preto, São Paulo - FAMERP

**Keywords:** epidemiology, facial bones, facial injuries.

## Abstract

**Abstract:**

Facial traumas are frequent in emergencies, and they require the diagnosis of fractures and associated lesions.

**Aim:**

To analyze epidemiological data concerning facial trauma care.

**Materials and Methods:**

Three hundred and fifty-five charts from patients with facial trauma treated by the Service of Otorhinolaryngology, from January 2002 to December 2008, were revised. The following data was collected: age, gender, etiology, anatomical localization of the fracture, associated injuries, alcohol consumption, treatment, and hospitalization.

**Study Design:**

A retrospective historical longitudinal study.

**Results:**

Most of the patients are young adult men (p<0.05) with a male:female ratio of 4:1(p<0.05). Interpersonal violence is the most prevalent cause of facial trauma (27.9%), followed by motor vehicle accidents (16.6%) (p<0.05). The mandible is the most prevalent facial bone fractured (44.2%), followed by nasal fracture (18.9%) (p<0.05). 41.1% of the patients consumed alcohol with a male:female ratio of 11.2:1 (p<0.05). Seventy-seven percent of the patients required surgical intervention (p<0.05) and 84.5% were hospitalized (p<0.05).

**Conclusion:**

Young male adults are the most prevalent victims of facial trauma, and interpersonal violence is responsible for the majority of the facial injuries. Most of the cases of facial trauma are associated with the consumption of alcohol. Further studies will be necessary to provide a clear understanding of the trends in the etiology of facial trauma.

## INTRODUCTION

All aspects regarding trauma have a great importance in the world today, being among the main causes of morbimortality. Each day, about 16,000 people die because of trauma^1^. Among the innumerous injuries seen in urban trauma centers, facial trauma is one of the most prevalent. Since it is the most exposed part of the body, and the one least protected, the face is the region which is most associated with other organs or systems in emergency centers^2^.

On the maxillofacial region, mandible and nose fractures are the most prevalent, followed by the zygomatic bone^3^,^4^. The epidemiology of facial fractures varies with type and lesion cause and severity, depending on the sample studied. Although the accidents caused by motor vehicles still represent the main cause of maxillofacial trauma in some developed countries. Recent data from these countries indicate that interpersonal violence has become another prevalent cause. The world trend in the reduction of maxillofacial lesions associated with automobile accidents is associated to the combination of better road conditions, modern safety systems installed to the vehicles, implementation of punishment to drunk drivers, lowering speed limits, increase demands as far as safety systems in vehicles is concerned and the need to use a safety belt^5^, ^6^, ^7^.

Fractures stemming from both etiologies, automobile accident and interpersonal violence involve patients in the age range between 20 and 29 years of age. Interpersonal violence frequently happens in homes, involving young men and having alcoholic beverages as the major contributing factor. The fracture sites are the result of the mechanism of injury. Interpersonal aggressors commonly aim at the mandible and/or the zygomatic bone because of their greater prominence on the facial anatomy, while automobile accidents tend to result in more complex fractures because of the high speed impact. In any way, in both groups the patients frequently require surgical intervention and hospitalization^8^, ^9^, ^10^.

Facial trauma stand out not only because of its importance, since they also bear emotional, functional and cosmetic repercussions, whether permanent or not, but also for representing about 7.4% to 8.7% of the medical care provided in emergency centers. About 80.7% of the patients are males. This is likely due to the fact that there are more men driving, practicing physical activities, and abusing drugs and/or alcohol before driving. Nonetheless, in the last decades there has been a growing number of trauma involving women, usually below 40 years of age. This is due to the behavioral changes women are going through in our society, with a larger number of them having jobs, the association between alcohol and driving and the practice of sports requiring more physical contact^11^, ^12^, ^13^, ^14^, ^15^, ^16^, ^17^.

Because of the high incidence and prevalence of facial trauma, it is necessary to have a clear understanding of the patterns of injuries which affect the face, and which can help in emergency care in order to provide for more adequate and effective treatment. Moreover, such epidemiological information may also be used in order to implement protocols guided towards implementing prevention programs^18^.

## OBJECTIVES

This paper aims at reporting the epidemiological data of patients with facial trauma seen by the medical team of the Department of Otorhinolaryngology and Head and Neck Surgery of a teaching hospital, between 2002 and 2008.

## MATERIALS AND METHODS

According to the regulations concerning Research with Human Beings, Resolution 196/96 from the Ministry of Health; the present investigation was approved by the Ethics Committee (CEP) of the institution (Report # 386/2009). This is a retrospective study, from a historical longitudinal cohort, for which we used the data obtained from reviewing the medical charts of 355 patients who were the victims of facial trauma, seen between 2002 and 2008. Because of this, it was not necessary to have the patients sign the Free and Informed Consent Form.

The following criteria were evaluated: age at the time of the accident, gender, accident type (automobile, interpersonal violence, car hit, fall, bicycle, motorcycle, ox heading, fire weapon, non-lethal weapon, occupational accident and “others” in which we include less frequent types of accident such as assault with an iron bar to the face, horse heading - horse riding, heading between people, sports - soccer and pool, ox or horse kick, metal objects against the patient, ox stepping, accident with tractor, door to the face, found unconscious and “not informed”), injury characteristics, associated injuries, use of alcohol and/or drugs (found through information from the patient or from the companion, police officers and/or physical exam), treatment type (surgical and/or conservative), need for hospitalization and/or in an intensive care unit.

In order to better characterize the sample, the age range was classified according to growth stages: childhood (2 to 10 years), adolescence (11 to 17 years), young adult (18 to 40 years), adult (41 to 65 years) and elderly (> 65 years)^19^, ^20^.

We calculated their mean values (M), percentages (%) and standard deviations (SD); and the results were expressed in, respectively, M (MSD) and % (%SD) and we used the Chi-square and/or Fisher's Test in order to compare the variables, when applicable.

## RESULTS

During the period of 2002 to 2008, 355 patients were affected by facial trauma, 283 men (79.7%, SD% =2.4) and 72 women (20.3%, SD% =4.7) with a statistically significant difference (p<0.05). As far as age is concerned, it varied between 7 and 89 years with a mean value of 28.8 years (MSD=1.7) for men; and from 5 to 83 years, with a mean value of 29.3 years (MSD=1.9) for women. According to the age range classification, the association with growth stages was statistically significant (p=0.043) because of the higher predominance of men in the young adult stage at the time of the trauma (Table 1).

**Table 1:** Patient distribution in relation to growth stages at the time of the trauma.

Growth Stages	Males (%)	Females (%)
Childhood (2 to 10 years)	7 (1,9)	5 (1,4)
Adolescence (11 to17 years)	28 (7,9)	8 (2,2)
Young adult (18 to 40 years)	203 (57,2)	44 (12,4)
Adult (41 a 65anos)	38 (10,8)	9 (2,6)
Elderly (> 65 years)	7 (1,9)	6 (1,7)
Total (%)	283 (79,7)	72 (20,3)

Of all the patients, 209 did not use alcohol or drugs (58.9%, SD%=3.4) and 146 were drunk or drugged at the time of the accident (41.1%, SD%=4.0) and from these drunk, 134 were men (91.8%, SD%=2.3) and 12 were women (8.2%, SD%=7.9); and this ratio was statistically significant (p<0.05).

Among the trauma mechanisms we found the following more frequent categories: interpersonal violence in 99 cases (27.9%, SD%=4.5) and car accidents in 59 cases (16.6%, SD%=5.2), and this difference was significant (p<0.05). Chart 1 shows the mechanisms which caused the facial trauma to the patients in this study.

**Graph 1:**
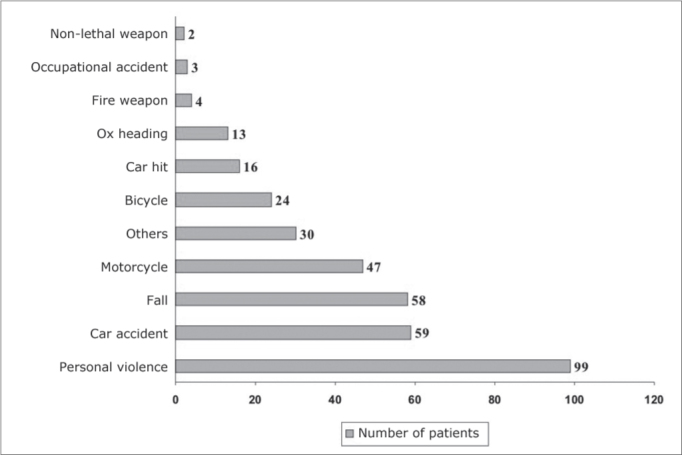
Distribution of the patients in relation to the trauma etiology.

**Graph 2:**
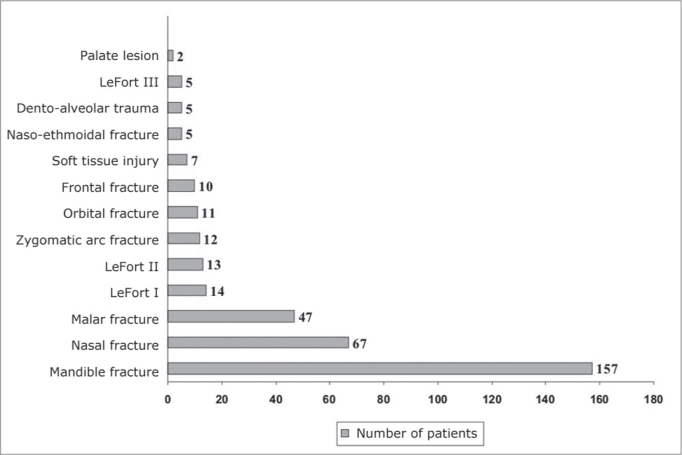
Distribution of the patients in relation to the primary anatomical sites of the injuries.

In relation to the diagnosis and primary anatomical site of the injuries, mandible and nasal fractures were the most frequent, affecting 157 (44.2%, SD%=3,9) and 67 (18.9%, SD%=4.7) patients, respectively; and such association was statistically significant (p<0.05). The other sites involved, as well as the number of patients involved are depicted on Graph 2. The anatomical sites of the mandible fractures happen on the following sites, per order of frequency: parasymphyseal (76%), condyle (45%), body (38%), angle (25%), symphyseal (13%), branch (10%) and coronoid process (3%).

We diagnosed 80 patients (22.5%, SD%=4.6) with associated injuries on the primary diagnosis (p<0.05), and the soft tissue injuries were the most frequent, affecting 30 patients (37.5%, SD%=8.8), followed by nasal fracture in 14 cases (17.5%, SD%=10.1), and such association was significant (p=0.020). Of the fifteen trauma mechanisms, soft tissue injuries happened in eight of them (72.7%, SD%=13.4), except on the ox heading, occupational accident and non-lethal weapon categories. Interpersonal violence and automobile accidents were the mechanisms which most often had associated injuries, with a frequency of: 23.75% (SD%=9.7) and 16.25% (SD%=10.2), respectively (Table 2).

**Table 2:** Trauma mechanisms distribution in relation to the associated injuries in the primary diagnosis.

	Soft tissue injuries	Nasal fracture	Alveolar trauma	Orbit fracture	Malar fracture	Mandibular fracture	Palate injury	LeFort I	LeFort II	Total n(%)
Interpersonal Violence	9	4	3	3	0	0	0	0	0	19(23,75)
Automobile Accident	5	3	1	1	1	2	0	0	0	13(16,25)
Fall	3	2	1	1	2	0	0	1	0	10(12,5)
Motorcycle	4	0	3	2	2	1	1	0	0	13(16,25)
Others	2	1	1	0	1	0	0	0	0	5(6,25)
Bicycle	2	2	2	1	1	0	0	0	0	8(10)
Car Hit	3	2	0	0	0	0	0	0	1	6(7,5)
Ox heading	0	0	1	0	0	0	0	0	0	1(1,25)
Fire arm	2	0	0	0	0	0	0	0	0	2(2,5)
Occupational Accident	0	0	0	1	1	0	0	0	0	2(2,5)
Non-lethal weapon	0	0	0	1	0	0	0	0	0	1(1,25)
Total n(%)	30(37,5)	14(17,5)	12(15)	10(12,5)	8(10)	3 (3,75)	1(1,25)	1(1,25)	1(1,25)	80(100)

Closed surgical treatment was necessary in 274 patients (77.2%, SD%=2.5) and conservative treatment in 81 patients (22.8%, SD%=4.6) (p<0.05). Three hundred patients (84.5%, SD%=2.0) needed to be hospitalized and 13 (3.7%, SD%=5.2) required ICU treatment (p<0.05).

The specific characteristics of each category which caused trauma in our sample are described below, by order of frequency, and the general characteristics are presented on Tables 2, 3 and 4.

**Table 3:** Trauma mechanisms distribution in relation to growth stages among the patients who drank alcoholic beverages.

	childhood (2 to 10 years)		adolescence (11 to 17 years)		young adult (18 to 40 years)		adult (41 to 65 years)		elderly (> 65 years)		Total n-146	
	M	F	M	F	M	F	M	F	M	F	M	F
Interpersonal violence	1	0	1	1	42	0	8	3	0	0	52	4
Automobile accident	0	0	3	0	15	0	3	2	0	0	21	2
Fall	0	0	2	0	13	1	4	1	0	0	19	2
Motorcycle	0	0	0	0	19	1	2	0	0	0	21	1
Others	0	0	1	0	5	1	2	0	0	0	8	1
Bicycle	0	0	0	0	3	1	2	0	0	0	5	1
Car hit	0	0	0	0	4	0	0	0	0	0	4	0
Ox heading	0	0	0	0	1	0	0	0	0	0	1	0
Fire arm	0	0	0	0	2	0	0	0	0	0	2	0
Occupational accident	0	0	0	0	0	0	0	0	0	0	0	0
Non-lethal weapon	0	0	0	0	0	1	1	0	0	0	1	1
Total n(%)	1(0,7)	0(0)	7(4,8)	1(0,7)	104(71,2)	5(3,4)	22(15,1)	6(4,1)	0(0)	0(0)	134(91,8)	12(8,2)

**Table 4:** Trauma mechanisms distribution in relation to the primary anatomical site of the injuries.

	Mandible	Nose	Malar	LeFort I	LeFort II	Zygomatic arch	Orbit	Frontal	Soft tissue injury	Nasoethmoidal	Alveolar	LeFort III	Palate	Total n(%)
Interpersonal violence	39	26	12	5	1	3	6	0	1	3	1	1	1	99 (27,9)
Automobile accident	25	8	8	4	5	3	0	2	1	1	1	1	0	59 (16,6)
Fall	26	12	10	1	1	2	3	0	2	0	1	0	0	58 16,3)
Motorcycle	27	7	5	2	3	0	1	1	0	0	0	1	0	47 (13,2)
Others	7	9	6	1	1	3	0	3	0	0	0	0	0	30 (8,5)
Bicycle	13	4	2	0	0	1	0	2	0	0	2	0	0	24 6,8)
Car hit	6	1	2	1	1	0	1	0	2	0	0	2	0	16(4,5)
Ox heading	9	0	2	0	1	0	0	0	0	1	0	0	0	13 (37)
Fire arm	3	0	0	0	0	0	0	0	0	0	0	0	1	4 (1,1)
Occupational accident	2	0	0	0	0	0	0	1	0	0	0	0	0	3 (0,8)
Non-lethal weapon	0	0	0	0	0	0	0	1	1	0	0	0	0	2 (0,6)
Total n(%)	157(44,2)	67(18,9)	47(13,3)	14(3.9)	13(3,7)	12(3,4%)	11(3,1)	10(2,8)	7(1,9)	5(1,4)	5(1,4)	5(1,4)	2(0,6)	355 (100)

### Interpersonal violence

Interpersonal violence was the most frequent etiology of the traumas found in the present study, predominating among the men - 83 cases (83.8%, SD%=4.0) and 16 women (16.2%, SD%=9.2). Age varied between 7 and 56 years, with a mean value of 29.1 years (MSD=1.1) for men; and 5 to 50 years, with a mean value of 23.0 years (MSD=2.8) for women. Fifty-two men (62.6%, SD%=6.7) and 4 women (25%, SD%=21.6) were drunk, and such ratio was significant (p=0.011). There was a predominance of drunk men among young adults - 42 (80.6%, SD%=6.1); nonetheless it is important to stress that this was the only category which had a drunk patient at a pediatric age (10 years) (Table 3). Violence was the category which most resulted in injuries and mandible fracture was the most frequent in 39 cases (39.4%, SD%=9.3) followed by nasal in 26 cases (26.3%, SD%=8.6) (Table 4). Among the injuries associated with facial trauma, soft tissue injuries were the most prevalent in 9 patients (47.4%, SD%=11.4) (Table 2). It is important to stress that in this category, among the 12 patients in the children and adolescents groups, 4 (33.3%, SD%=23.5) children suffered interpersonal violence, which did not happen in the elderly population of our sample.

### Automobile Accident

Automobile accidents were more frequent in men - 46 cases (78%,SD%=6.1) compared to 13 women (22%, SD%=11.4). Age varied between 16 and 64 years, mean age = 29.6 years (MSD=1.8), for men and 14 to 50 years, mean of 30.6 years (MSD=3.8), for women. Of these, 21 men (45.6%, SD%=10.8) and 2 women (15.4%, SD%=25.5) were drunk (p=0.059). Fifteen drunk men (71.4%, SD%=11.6) were young adults (Table 3). Mandible fracture was also the most prevalent injury in 25 cases (42.4%, SD%=9.9) (Table 4). Soft tissue injury was the most frequent associated injury in 5 cases (38.5%, SD%=21.8) (Table 2).

#### Falls

Thirty-eight men (65.5%, SD%=7.7) and 20 women (34.5%, SD%=10.6) fell. The prevalence peak varied between 7 and 89 years, mean of 32.1 years (MSD=3.1) for men; and 5 to 83 years, mean of 37.7 years (MSD=5.5) for women. Of these, 19 men (50%, SD%=11.4) and 2 women (10%, SD%=21.2) were drunk, and this difference was statistically significant (p=0.004). Thirteen men were (68.4%, SD%=12.8) drunk and this was most prevalent among young adults (Table 3). Once again, mandible fracture was the most frequent injury in 26 patients (44.8%, SD%=9.7) (Table 4). Soft tissue injury was the most prevalent of facial trauma associated injuries in 3 patients (30%, SD%=26.4) (Table 2). In this category, non-drunk patients also fell, and most of them were in the extremes of age, that is 5 in children (41.7%, SD%=22) and 6 senior citizens (46.1%, SD%=20.3).

### Motorcycle

Accidents with motorcycles happened to 40 men (85.1%, SD%=5.6) and 7 women (14.9%, SD%=13.4). Age varied between 13 and 72 years, mean value of 27.6 years (MSD=1.7) for men; and 16 to 34 years, mean value of 24.8 years (MSD=2.6) for women. Of these, 21 men (52.5%, SD%=10.8) and 1 woman (14.2%, SD%=34.9) were drunk (p=0.102). Among the drunken men, 19 (90.5%, SD%=6.7) were young adults (Table 3). The mandible fracture was the most frequent in 27 cases (57.4%, SD%=9.5) (Table 4). Among the facial trauma associated injuries, soft tissue was the most prevalent one in 4 cases (30.7%, SD%=23) (Table 2).

### Others

In this category we included the low prevalence accidents; nonetheless, frequent in the region where the study was carried out - a rural area with rodeos and this sort of parties: assault with an iron bar to the face (1 case, 3.33%, SD%=17.9 ), hoarse heading - horse riding (1 case, 3.33%, SD%=17.9), heading between persons (4 cases, 13.37%, SD%=17.0), ox kick (3 cases, 10%, SD%=17.3) or horse kick (1 case, 3.33%, SD%=17.9), found unconscious (1 case, 3.33%, SD%=17.9), sports [soccer (1 case, 3.33%, SD%=17.9), pool ball (1 case, 3.33%, SD%=17.9), pool stick (1 case, 3.33%, SD%=17.9), sports in general (3 cases, 10%, SD%=17.3)], metal objects against the patient (1 case, 3.33%, SD%=17.9), ox step over (3 cases, 10%, SD%=17.3), door against the face (1 case, 3.33%, SD%=17.9), hook fall against the face (1 case, 3.33%, SD%=17.9), tractor - head compression (1 case, 3.33%, SD%=17.9) and not informed (6 cases, 20%, SD%=16.3).

Men were the most affected, making up a total of 27 (90%, SD%=5.7) cases and only 3 were women (10%, SD%=17.3). Age varied between 7 and 43 years, mean of 24.8 years (MSD=1.8) for men; and 22 to 36 years, mean of 28.0 years (MSD=4.2) for women. Of these, 8 men (29.6%, SD%=16.1) and 1 woman (33.3%, SD%=47.1) were drunk (p=1.000). Among the male patients who were drunk, most (5) were young adults (62.5%, SD%=21.6) (Table 3). Nasal fracture was the most frequent in 9 patients (30%, SD%=15.2) (Table 4). Soft tissue injuries happened to 2 patients (40%, SD%=34.6) as the most frequent injury associated to facial trauma (Table 2).

#### Bicycle

The trauma caused by bicycle accidents affected 18 men (75%, SD%=10.2) and 6 women (25%, SD%=17.6). Age varied between 14 and 54 years, mean value of 25.7 years (MSD=2.8) for men and 13 to 33 years, mean of 21.3 years (MSD=4.2) for women. Among these, 5 men (27.8%, SD%=20.0) and 1 woman (16.6%, SD%=37.2) were drunk (p=1.000). Of the drunken patients, 3 (60%, SD%=28.2) were young adults (Table 3). The mandibular fracture was the most frequent in 13 patients (54.2%, SD%=13.8) (Table 4). Soft tissue injuries were equally associated with nasal fractures and alveolar trauma, with two cases each (25%, SD%=30.6) (Table 2).

#### Hit by a car

The victims of car hits were 12 men (75%, SD%=12.5) and 4 women (25%, SD%=21.6). The age range for men varied between 12 and 72 years, mean of 33.5 years (MSD=6.0), and for women it varied between 19 and 63 years, mean of 31.2 years (MSD=10.6). From these, 4 patients (33.3%, SD%=23.5) - only young adult men, were drunk (Table 3). The mandible fracture was the most frequent injury, affecting 6 patients (37.5%, SD%=19.7) (Table 4). Among the injuries associated with facial trauma, soft tissue injuries were the most frequent in 3 patients (50%, SD%=28.8) (Table 2).

#### Ox heading

Twelve men (92.3%, SD%=7.6) and one woman (7.7%, SD%=26.6) suffered ox heading trauma. Age varied between 13 and 35 years, mean of 24 years (MSD=1.7), for men and the only woman at the time of the trauma was 29 years old. Of these, only 1 man (8.3%, SD%=27.5), a young adult was drunk (Table 3). Mandible fracture was the most frequent in 9 patients (69.2%, SD%=15.3) (Table 4). Alveolar trauma was the only injury associated with the facial trauma, and it happened to 1 patient only (100%) (Table 2).

#### Fire Arm

Three men (75%, SD%=25) and 1 woman (25%, SD%=43.3) suffered injuries caused by fire arms. Their age varied between 32 and 34 years, mean value of 32.6 years (MSD=0.64), for men and the only patient was 27 years old, at the time of the trauma. Only two male patients (66.7%, SD%=33.3) were drunk, both were young adults (Table 3). Mandible fracture was the primary fracture in 3 patients (75%, SD%=25) (Table 4). Two patients had soft tissue injuries (100%) and it was the only type of injury associated with facial trauma (Table 2).

#### Occupational accident

Only three male patients (100%) suffered occupational injuries. Their ages varied between 24 and 38 years, mean of 29 years (MSD=4.5), and they were all young adults (100%). None of these patients were drunk (Table 3). Mandible fractures happened in 2 patients (66.7%, SD%=33.3) (Table 4). As facial-trauma-associated injuries, orbit and malar fractures happened equally to one case each (50%, SD%=50) (Table 2).

#### Non-lethal weapon

Two patients, one man and 1 woman (50%, SD%=50), suffered non-lethal weapon injuries, and they were both drunk (100%). The male patient was 43 years old (Table 3) at the time of the accident, having a frontal trauma as the primary injury (Table 4) and orbit fracture as the only associate injury (Table 2). The female patient was, at the time of the accident, 24 years (Table 3), there was only soft tissue injury (Table 4); there were no facial-trauma-associated injuries (Table 2).

## DISCUSSION

The continuous communication of data associated with facial trauma epidemiology is extremely important in order to provide the necessary information for the development and evaluation of preventive action aiming at reducing the incidence of facial injuries. Because of this, the present study assessed the profile of the patients who were victims of facial trauma, seen by the medical team of the ENT-HNS Department of a teaching tertiary care hospital in the State of São Paulo.

The facial injuries affected mostly male patients, in a ratio of approximately 4:1 and this ratio was within the ranges described in the literature, which varied between 2.6:1 to 11.8:^1^,^7^,^13^, ^14^, ^15^, ^16^, ^17^, ^18^,^21^, ^22^, ^23^, ^24^. This high vulnerability of men to most types of trauma can be associated to the fact that in our society men has more freedom to have jobs and is more engaged in high risk activities, thus being more vulnerable to accidents. Nonetheless, there has been a world trend of increase in women being more exposed to the risk factors which cause facial trauma, as found in this study, in which only the occupational accident category did not have any woman - indicating that in all the other categories there were women. The lack of occupational injuries in women may be due to the lower number of women having jobs and/or the very characteristic of females being more careful in any activity they participate in.

The age distribution of the patients with facial trauma in the present study corresponds to the data in the literature. The young adult patients (18 to 40 years old) were the most prevalent from both genders in our study; however with a statistically significant predominance of men when we consider all the mechanisms causing facial trauma. Young adults usually have greater physical skills and mobility and are more often present in risky situations, and they also drink more alcoholic beverages, as it was found in the present study^7^,^13^, ^14^, ^15^, ^16^, ^17^, ^18^,^22^,^25^,^26^.

Approximately 92% of the patients who were drunk or drugged at the time of the accident were men, at an 11.2:1 ratio, and this result was found in all the categories of trauma established in the present study, except for occupational accidents, very likely because of the laws in effect which rule occupational safety. There is a direct correlation between the amount of alcohol drank and the degree of the accidents, on its most varied ways, which cause facial trauma in all cultures and societies. With the increase in alcohol intake, there is a risk for the individual committing the act or suffering it. Each factor affects and increases the other^9^,^27^.

Of all the categories responsible for facial trauma in the present study, interpersonal violence (27.9%) was the most prevalent, and when compared to the second most frequent category, automobile accidents (16.6%), this association was statistically significant. These results are in accordance with the literature^7^,^13^, ^14^, ^15^, ^15^, ^17^, ^18^,^28^, ^29^, ^30^, except in some studies in which automobile accidents were the most prevalent^12^,^15^,^16^,^22^,^31^,^32^. Interpersonal violence has become one of the major problems of many areas and the increase in its rates has been associated with the ingestion of alcohol and/or the use of drugs^9^,^27^, according to the present study. The reported studies which still have not shown this trend towards an increase in the rates of interpersonal violence may have been so because of the region where the study was carried out and/or the inclusion of patients who suffered traumas during the 80's or the 90's when the use of safety equipment was not mandatory in cars, especially not in Brazil.

Although fall was the third category in the order of frequency, it was the one which proved to be the most important trauma mechanism in age extremes, stressing that 46.1% of the victims were above 65 years and 41.7% of the victims were children, and none of them were under the influence of alcohol. Interpersonal violence represented about 33.3% of the pediatric victims, and there were no injuries to the elderly. Aging is characterized by the gradual reduction in biological functions, with multiple sensory deficits, visual and auditory among them, changes in cognition and memory, and bone and muscle disorders increasing the risk of falls; while in children, a number of factors cause falls, since it is not only neurological centers which are involved, but also all those associated with balance and movement which are still being developed, and children do not know the difference between dangerous actions and the safe ones^14^,^16^,^33^, ^34^, ^35^, ^36^.

Mandible (44.2%) and nasal (18.9%) fractures were the most prevalent injuries found in our study. Mandible fracture was the one which occurred more often in all trauma categories, except for non-lethal weapons and “others”. Nasal fractures, in a second place of prevalence, when associated with mandibular fractures, were frequent in the categories of interpersonal violence, automobile accidents, falls and motorcycle and bicycle accidents. In the “others” category, nasal fracture was the one which occurred more often when compared to the mandible. On the maxillofacial region, mandible fracture is the most prevalent, followed by the zygomatic bone^3^,^4^. The epidemiology of facial fractures varies with the type, severity and cause of the injury, depending on the population studied. Although the accidents by motor vehicles are still the main cause of maxillofacial trauma in some developed countries, recent study data, from these same countries, indicate that interpersonal violence has become another prevalent cause^5^, ^6^, ^7^, as found in the present study.

According to the literature, assaults are usually responsible for mandible body and mandible angle fractures, automobile accidents are responsible for condyle fractures and mandibular body or problems on the condyle and symphysis and, the accidents with motorcycles, most of the times without wearing helmets, are responsible for fractures on the body, symphysis, parasymphysis and condyle^13^,^15^. The results from the present study are in agreement with the literature, considering the most prevalent fractures on parasymphysis, condyle and body, arising from these three facial trauma causing mechanisms, which in the present study were also the ones which happened more often.

Some studies report that the nose is the main site of fractures on the face, followed by the zygomatic bone because it has a central position on the face and it is an easy structure to be fractured because of the thin thickness of the nasal bones^17^,^22^,^30^. However, in the present study, mandible fractures were the most prevalent in most of the categories, and such result is in agreement with the literature^12^, ^13^, ^14^, ^15^, ^16^,^31^,^37^.

Patients with nasal fractures can have associated injuries with varied degrees of complexity and severity. Among the 80 (22.5%) injuries associated with the primary site of the facial trauma, in the present study, soft tissue injuries were the most prevalent (37.5%) followed by nasal fractures (17.5%) and these results are statistically significant. Very few studies report a high frequency of these injuries associated to facial trauma, in which the incidence varies from 10.3% to 51.6%, and the present study is within this range^14^,^15^,^38^, ^39^, ^40^, ^41^, but they do not report on which is the causing mechanism. In the present study, the associated injuries most often came from interpersonal violence, automobile and motorcycle accidents; in fact, the same mechanisms responsible for the greater frequency of mandibular fractures. The occurrence of associated injuries can be changed in relation to the geographic region involved, considering the life habits of the different populations. In any way, the nature and frequency of these injuries continue to be significant, according to what was found in this study; and the diagnosis and multiprofessional care are fundamental whenever necessary.

Hospitalization was necessary in 84.5% of the patients and 3.7% required care in the ICU. There is no literature data about the results found, only references as to the type of treatment, whether surgical or conservative. In the present study, surgical treatment was required in 77.2% of the cases, when compared to the conservative one, and this data was statistically significant. The indications for surgery included simple or complex factures with skull involvement, associated injuries, bruises and sutures for scalp laceration. The surgical approach is in agreement with the present protocol from the Department of Otorhinolaryngology and Head and Neck Surgery, following the world trend, including reduction, immobilization and fixation to the proper anatomical position of the fragments which shifted in relation to the other bones and the skull base. Non-shifted fractures are treated conservatively and the shifted ones are treated by open reduction and rigid internal fixation with miniplates. Facial and/or vertical rigid fixation of a facial fracture helps in the support and strength of masticatory function and conservative methods for non-shifted fractures provide acceptable cosmetic and functional results^3^,^13^,^18^,^25^,^42^, ^43^, ^44^, ^45^.

The epidemiological data associated with the facial trauma depend on the demographic data from the population studied, which include the geographic region, socio-economic situation, time factors such as the season of the year, which can influence the type and frequency of injuries in the population. In a logistics regression analysis we noticed that the maxillofacial fractures are not associated with the job type, but rather to the person's educational level^45^.

Although in the present study, in all trauma type categories there was a predominance of men, women can also be victims of trauma, especially interpersonal violence, on all its forms, and most of the times there are large amounts of alcoholic beverages involved. The World Health Organization reports that violence is responsible for the high health care costs and for about 73,000 deaths/year in Europe^27^.

In 1998, The British Association of Oral and Maxillofacial Surgeons organized an educational forum called “Save your Face”. Such educational program involved twohundred surgeons from the British Oral and Maxillofacial Hospital who visited young 13 and 14 year-old students at their schools in order to educate them on the harmful effects of a high ingestion of alcoholic beverages and its association with facial injuries^46^.

Alcohol ingestion prevention programs and educational policies should not be limited to the young only, but rather involve all stages of growth, from childhood to old age, considering their importance and order to reduce, in all age ranges, the incidence of alcohol-induced facial trauma.

## CONCLUSION

Facial trauma happened most frequently to young adult men, arising from interpersonal violence and automobile accidents. The most common anatomical sites affected by primary fractures on the face were the mandible and the nose, and soft tissue injuries were the most common associated lesions.

The use of drugs or alcohol intake was the most important associated factor in all the trauma categories, except in the occupational accident category. There is an urgency and great need to install educational and preventive actions in order to reduce the intake of alcoholic beverages and/or drugs in order to reduce the incidence of alcohol-induced facial trauma across all ages.

The continuous communication of data associated to the epidemiology of facial trauma is of the utmost importance because of the change tendency in cause mechanisms, which enables the creation of new guidelines to prevent these injuries.

## References

[1] Krug EG, Sharma GK, Lozano R (2000). The global burden of injuries. Am J Public Health..

[2] Alvi A, Dohert T, Lewen G (2003). Facial fractures and concomitant injuries in trauma patients. Laryngoscope..

[3] Hogg NJ, Stewart TC, Armstrong JE, Girotti MJ (2000). Epidemiology of maxillofacial injuries at trauma hospital in Ontario, Canada, between 1992 and 1997. J Trauma..

[4] Brasileiro BF, Passeri LA (2006). Epidemiological analysis of maxillofacial fractures in Brazil: a 5-year prospective study. Oral Sur Oral Med Oral Pathol Oral Radiol Endod..

[5] Ogundare BO, Bonnick A, Bayley N (2003). Pattern of mandibular fractures in an urban major trauma center. J Oral Maxillofac Surg..

[6] Adeyemo WL, Ladeinde AL, Ogunlewe MO, James O (2005). Trends and characteristics of oral and maxillofacial injuries I Nigeria: a review of the literature. Head Face Med..

[7] Lee KH, Snape L, Steenberg LJ, Worthington J (2007). Comparasion between interpersonal violence and motor vehicle accidents in the aetiology of maxillofacial fractures. ANZ J Surg..

[8] Olasoji HO, Tahir A, Arotiba GT (2002). Changing picture of facial fractures in northern Nigeria. Br J Oral Maxillofac Surg..

[9] Shaphiro AJ, Johnson R, Miller SF, McCarthy MC (2001). Facial fractures in a level I trauma centre: the importance of protective devices and alcohol abuse. Injury..

[10] Yokoyama T, Motozawa Y, Sasaki T, Hitosugi M (2006). A retrospective analysis of oral and maxillofacial injuries in motor vehicle accidents. J Oral Maxillofac Surg..

[11] Mackenzie EJ (2000). Epidemiology of injuries: current trends and future challenges. Epidemiol Rev..

[12] Mota VC, Aguiar EG, Dutra CEA (2001). Levantamento sobre os atendimentos de trauma facial. RGO..

[13] Chrcanovic BR, Freire-Maia B, Souza LN, Araújo VO, Abreu MHNG (2004). Facial fractures: a 1-year retrospective study in a hospital in Belo Horizonte. Braz Oral Res..

[14] Wulkan M, Parreira Jr JG, Botter DA (2005). Epidemiologia do trauma facial. Rev Assoc Med Bras..

[15] Martini MZ, Takahashi A, Oliveira Neto, HG, Carvalho Júnior JP, Cúrcio R, Shinohara EH (2006). Epidemiology of mandibular fractures treated in a brazilian level I trauma public hospital in the city of São Paulo, Brazil. Braz Dent J..

[16] Montovani JC, Campos MP, Gomes MA, Moraes VRS, Ferreira FD, Nogueira EA (2006). Etiologia e incidência das fraturas faciais em adultos e crianças: experiência em 513 casos. Braz J Otorhinolaryngol..

[17] Macedo JL, Camargo LM, Almeida PF, Rosa SC (2008). Perfil epidemiológico do trauma de face dos pacientes atendidos no pronto-socorro de um hospital público. Rev Col Bras Cir..

[18] Rajendra PB, Mathew TP, Agrawal A, Sabharawal G (2009). Characteristics of associated craniofacial with head injuries: An experience with 100 cases. J Emerg Trauma Shock..

[19] Estatuto do Idoso. Lei N^o^ 10.741, de 1° de Outubro de 2003. Série fontes de referência. Legislação: n^o^ 53. 42 p. ISBN:85-7365-345-0.

[20] Murahovski J (2003). Pediatria: Diagnóstico + Tratamento..

[21] Hächl O, Tuli T, Schwabegger A, Gassner R (2002). Maxillofacial trauma due to work-related accidents. Int J Oral Maxillofac Surg..

[22] Sobreira T, Vieira JAO, Lobo AR, Wanderley JNB, Costa LJ (2002). Prevalência de traumatismos bucomaxilofaciais em João Pessoa - Brasil. Rev Bras Cien Saúde..

[23] Al Ahmed HE, Jaber MA, Abu Fanas SH, Karas M (2004). The pattern of maxillofacial fractures in Sharjah, United Arab Emirates. A review of 230 case. Oral Surg Oral Med Oral Pathol Oral Radiol Endod..

[24] Zargar M, Khaji A, Karbakhsh M, Zarei MR (2004). Epidemiology study of facial injuries during a 13 month of trauma registry in Tehran. Indian J Med Sci..

[25] Ansari MH (2004). Maxillofacial fractures in Hamedan province Iran: a retrospective study (1987-2001). J Craniomaxillofac Surg..

[26] Rodrigues FH, Miranda ES, Sousa VEM, Castro VM, Oliveira DRF, Leão CEG (2006). Avaliação do trauma bucomaxilofacial no Hospital Maria Amélia Lins da Fundação Hospitalar do estado de Minas Gerais. Rev Soc Bras Cir Plast..

[27] World Health Organization (WHO). Global Status Report on Alchool 2004. Geneva: WHO, 2004.

[28] Valente ROH, Souza LCM, Antonini SV, Glock L, Nisa-Castro-Neto W (2003). Epidemiology of mandibular fractures assisted at Santa Casa de Misericórdia de São Paulo Hospital (HSCSP) between 1996 and 1998. Rev Bras Cir Period..

[29] Macedo JS, Camargo LM, Almeida PF, Rosa SC (2007). Mudança etiológica do trauma de face de pacientes atendidos no pronto socorro de cirurgia plástica do Distrito Federal. Rev Soc Bras Cir Plast..

[30] Pereira MD, Kreniski T, Santos RA, Ferreira LM (2008). Trauma craniofacial: perfil epidemiológico de 1223 fraturas atendidas entre 1999 e 2005 no Hospital São Paulo - UNIFESP - EPM. Rev Soc Bras Cir Craniomaxilofac..

[31] Tussi R, Stefenon M, Tussi Junior R, Avila GV (2000). Fraturas da face. Rev Med Hosp São Vicente de Paulo..

[32] Gabrielli MAC, Gabrielli MFR, Marcantonio E, Hochuli-Vieira E (2003). Fixation of mandibular fractures with 2,0 mm miniplates. Review of 191 cases. J Oral Maxillofac Surg..

[33] Shaikh ZS, Worrall SF (2002). Epidemiology of facial trauma in a sample of patients aged 1-18 years. Injury..

[34] Bulut M, Koksal O, Lorkmaz A, Turan M, Ozgue H (2006). Childhood falls: Characteristics, outcome, and comparasion of the injury severity score and new injury severity score. Emerg Med J..

[35] McGeehan J, Shields BJ, Wilkins JR 3rd, Ferketich AK, Smith GA (2006). Escalator-related injuries among children in the United States, 1990-2002. Pediatrics..

[36] Güzel A, Karasalihoglu S, Küçükugurluorglu Y (2007). Evaluation of the fall-related trauma cases applied to our pediatric emergency department. Ulus Travma Acil Cerrahi Derg..

[37] Kelley P, Crawford M, Higuera S, Hollier LH (2005). Two hundred ninety-four consecutive facial fractures in an urban trauma center: lessons learned. Plast Reconst Surg..

[38] Gwyn PP, Carraway JH, Horton CE, Adamson JE, Mladick RA (1971). Facial fractures-associated injuries and complications. Plast Reconst Surg..

[39] Olson RA, Fonseca RJ, Zeitler DL, Osbon DB (1982). Fractures of the mandible: a review of 580 cases. J Oral Maxillofac Surg..

[40] Ellis E 3rd, Moss KF, el-Attar A (1985). Ten years of mandibular fractures: An analysis of 2137 cases. Oral Surg Oral Med Oral Pathol..

[41] Lim LH, Lam LK, Moore MH, Trott JA, David DJ (1993). Associated injuries in facial fractures: review of 839 patients. Br J Plast Surg..

[42] Stanley RB (1995). Buttress fixation with plates. Operat Tech Otolaryngol Head Neck Surg..

[43] Fasola AO, Nyako EA, Obiechina AE, Arotiba JT (2003). Trends in the characteristics of maxillofacial fractures in Nigeria. J Oral Maxillofac Surg..

[44] Motamedi MH (2003). Na assessment of maxillofacial fractures: A 5-years study of 237 patients. J Oral Maxillofac Surg..

[45] Ribeiro MF, Marcenes W, Croucher R, Sheiham A (2004). The prevalence and causes of maxillofacial fractures in patients attending accident and emergency departments in Recife-Brazil. Int Dent J..

[46] Hutchison IL, Magennis P, Shepherd JP, Brown AE (1998). The BAOMS United Kingdom survey of facial injuries part 1: aetiology and the association with alcohol consumption. Br J Oral Maxillofac Surg..

